# The Importance of Left Ventricular Outflow Tract and Mid-Ventricular Gradients in Stress Echocardiography: A Narrative Review

**DOI:** 10.3390/jcm12165292

**Published:** 2023-08-14

**Authors:** Carlos Cotrim, Eszter Dalma Palinkas, Nuno Cotrim

**Affiliations:** 1 Heart Center do Hospital da Cruz Vermelha, 1500-048 Lisboa, Portugal; 2 Cardiovascular Unit (UCARDIO), 2350-325 Riachos, Portugal; 3 Hospital Particular do Algarve, Gambelas, 8005-226 Faro, Portugal; 4 Doctoral School of Clinical Medicine, University of Szeged, 6720 Szeged, Hungary; palinkaseszti@hotmail.com; 5 Santarém Hospital, 2005-177 Santarém, Portugal; nuno_cotrim1@hotmail.com

**Keywords:** intraventricular gradients, exercise echocardiography, angina, athletes, hypertrophic cardiomyopathy, tiredness with normal systolic function, syncope or dizziness related to exercise

## Abstract

This review aims to serve as a guide for clinical practice and to appraise the current knowledge on exercise stress echocardiography in the evaluation of intraventricular obstruction in HCM, in patients with cardiac syndrome X, in athletes with symptoms related to exercise, and in patients with normal left ventricular systolic function and exercise-related unexplained tiredness. The appearance of intraventricular obstruction while exercising is considered rare, and it usually occurs in patients with hypertrophy of the left ventricle. The occurrence of intraventricular obstruction when exercising has been evidenced in patients with hypertrophic cardiomyopathy, athletes, patients with cardiac syndrome X, patients with syncope or dizziness related to exercise, and patients with dyspnea and preserved ejection fraction. The clinical significance of this observation and the exercise modality that is most likely to trigger intraventricular obstruction remains unknown. Supine exercise and lying supine after exercise are less technically demanding, but they are also less physiologically demanding than upright exercise. Importantly, in everyday life, human beings generally do not become supine after exercise, as takes place in post-exercise treadmill stress echocardiograms in most echocardiography labs. The presence of induced intraventricular obstruction might be considered when patients have exercise-related symptoms that are not understood, and to assess prognosis in hypertrophic cardiomyopathy.

## 1. Introduction and Aims

We have been performing exercise stress echocardiography (SE) with the evaluation of cardiac function while exercising on a treadmill for more than 20 years. This method has been published [1] and is also used by other groups [2,3]. The assessment of exercise-induced intraventricular gradients with exercise echocardiography is increasingly used in clinical practice in the evaluation of patients with hypertrophic cardiomyopathy (HCM), in part due to the current guidelines’ recommendation to estimate the presence and increasing severity of obstruction with exercise [4]. The search for intraventricular gradients as an explanation for exercise-related symptoms in patients who have no structural heart disease is also gaining space in clinical practice. The correlation between exercise-elicited symptoms and intraventricular gradients has given us the opportunity to provide this group of patients with appropriate treatment. Recently, intraventricular gradients have been postulated as a possible mechanism in the development of apical aneurysms, and arrhythmias or angina in apical hypertrophic cardiomyopathy [5]. The present narrative review aims to appraise the knowledge in this field and to serve as a guide for clinical practice, overviewing exercise stress echocardiography in the evaluation of obstruction in HCM, patients with angina and normal coronary arteries, athletes with symptoms related to exercise, and patients with normal ejection fraction and unexplained tiredness related to efforts. 

## 2. Methods

We provide an overview of the pathophysiology of intraventricular obstruction induced by exercise, highlighting its determinants: preload, contractility, and gradient. We describe the main signs of dynamic obstruction on echocardiographs. We describe how to quantify the obstruction, and provide a discussion on the anatomic and functional correlates along with the outcome data of that evaluation. We give some tips and tricks for better use of this data in clinical practice, as well as how to report the findings, and how clinical guidelines and recommendations suggest we use it. 

### 2.1. Preload, Contractility, and Gradient

Normal cardiac function centers on producing the required cardiac output, which is achieved through a combination of the stroke volume and the heart rate. A complete cardiac cycle is composed of the diastole and the systole. Ventricular diastole involves an active process of myocardial relaxation, which creates a pressure gradient between the left atrium and the left ventricle, resulting in the opening of the mitral valve and forward blood flow. The gradients underlying the promotion of diastolic function and ventricular filling are also influenced by ventricular compliance. At the end of diastole, the contraction of the atrium increases the left atrium pressure and causes a new increase in the gradient, promoting blood flow to the left ventricle [6]. The preload is completed at this point. The diastolic gradients in the left ventricle occur between the left ventricle base near the mitral valve and the apex and between the apical zone and the left ventricular outflow tract (LVOT) [7]. These diastolic gradients seem to have an important role in left ventricular (LV) filling and emptying [8]. In clinical practice, invasive measurements of intraventricular pressures in the left ventricle and other cardiac chambers using fluid-filled catheters are inaccurate for assessing exceptionally low gradients and pressures. Instead, color Doppler M-mode measurements are more accurate for this kind of evaluation [9]. The intraventricular pressure gradients between the ventricular apical zone and the left ventricle outflow tract zone have been demonstrated to be strongly correlated with peak trans-mitral flow, early ventricular filling, and stroke volume [10]. These facts demonstrate that intraventricular gradients are relevant to normal filling and emptying, and especially to diastolic and systolic function. A typical pattern of systolic intraventricular pressure gradients has been verified within the left ventricle from 3.7 ± 0.4 mm Hg at the beginning of the systole between the LV apex and the LVOT and from 3.9 ± 0.5 mm Hg in the late systole between outflow tract and the apex [11]; however, the significance of this gradient is unclear.

The intraventricular pressure gradients between the apical zone and the outflow tract of the systemic ventricle are due to the active forces of the contracting ventricle and are related to the inotropic state [12,13]. When outflow obstruction is absent, the peaks of intraventricular gradients during systole are attained early and are provoked by impulsive forces [14]. The intraventricular pressure gradients increase with exercise [8] and adrenergic stimulus [13], and decrease with beta-blocker use [15]. 

Small intraventricular gradients are a common phenomenon of normal cardiac function. Three mechanisms explain their augmentation with exercise: an augmentation in the non-obstructive and physiological gradients; obstruction at the end of systole related to ventricular cavity obliteration; and obstruction at mid-systole caused by systolic anterior motion (SAM) of the mitral valve, causing limitation to ejection [16]. HCM is the most frequent cause of a dynamic intraventricular gradient, but certainly not the only one.

### 2.2. The Main Sign of Dynamic Obstruction

**Figure 1:**
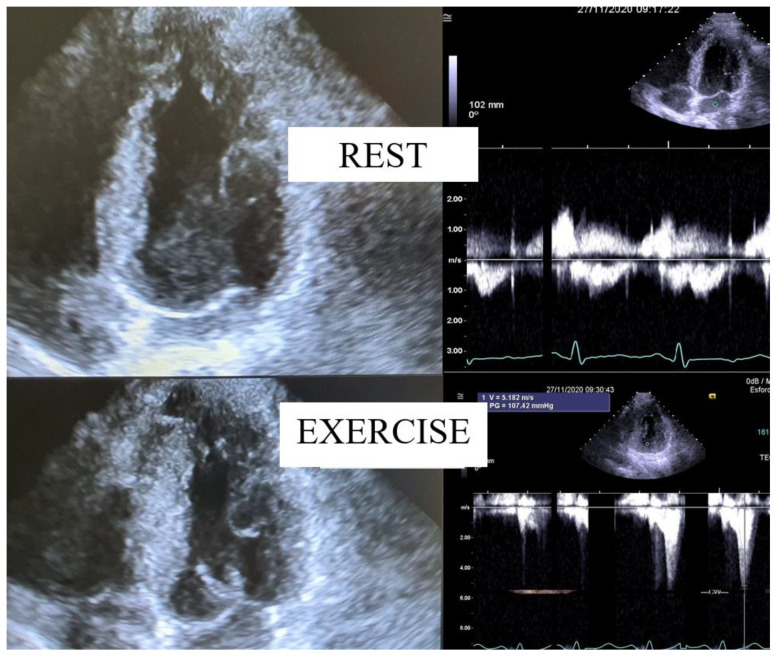
Systolic anterior motion (SAM) and an intraventricular gradient in a patient without hypertrophic cardiomyopathy (HCM), a 27-year-old soccer player with severe dizziness from exercise.

The intraventricular gradient is first evaluated with a color Doppler and a pulsed Doppler wave traveling from the LV apex to the LVOT in an apical four- or five-chamber view. Then, with the use of continuous Doppler, a typical “dagger-shaped” envelope is seen with late-peaking morphology (Figure 1). 

The main sign of intraventricular dynamic obstruction is present in most cases, and when SAM occurs (Figure 1), the configuration of the ventricular cavity or of the mitral valve apparatus is altered. Notably, a significant number of patients with intraventricular gradients [15,17] have mid-ventricular (MV) obstruction without SAM. 

### 2.3. Titration of Obstruction

The evaluation of the obstruction is performed with continuous-wave Doppler. Significant LVOT or MV obstruction is defined as a peak gradient greater than or equal to 30 mm Hg [18]. In one study [19], one in three healthy young subjects developed an LVOT gradient with peak velocities greater than 3 m/s and were considered normal. However, further studies are needed to better clarify the clinical significance of these gradients [20], although it has been demonstrated that intraventricular gradients can be caused by maneuvers that change loading conditions in structurally normal hearts [21].

### 2.4. Anatomic and Functional Correlates

**Figure 2:**
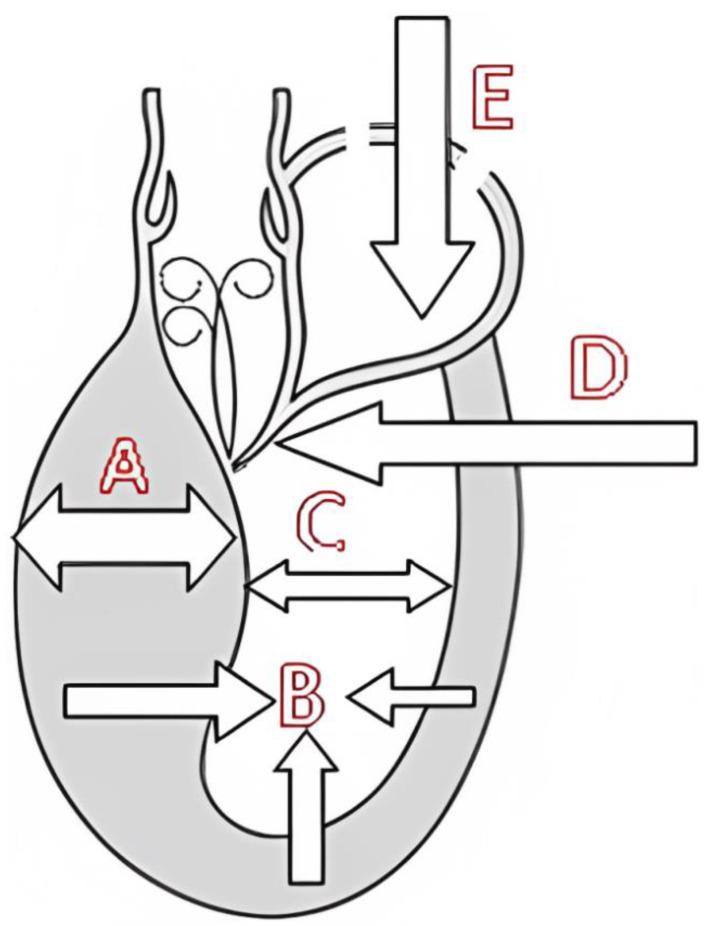
Mechanisms predisposing patients to left ventricular outflow tract (LVOT) gradient induction. A—Left ventricular (LV) hypertrophy, particularly of the basal septal segment (HCM, hypertension, storage disease). B—LV hypercontractility (moderate tachycardia). C—Small LV cavity (HCM, children, women, dehydration). D—Prolonged/thickened mitral leaflet(s). E—Reduced LV preload (dehydration, diuretics, vasodilators, hemodialysis, fever, septic shock, at the end of cardiac surgery when weaning extracorporeal circulation, orthostatic position) [3].

The factors that determine the occurrence of the LVOT gradient are displayed in Figure 2 [3]. 

**Figure 3:**
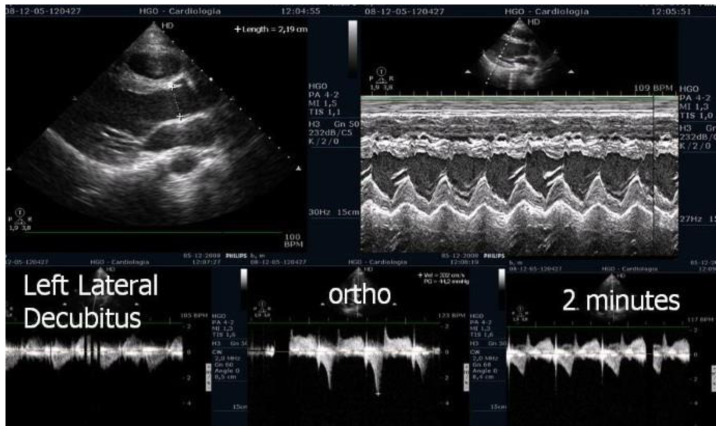
Echocardiography before exercise in a symptomatic athlete in the left lateral decubitus position and in the orthostatic position before and at the beginning of exercise [3].

**Figure 4:**
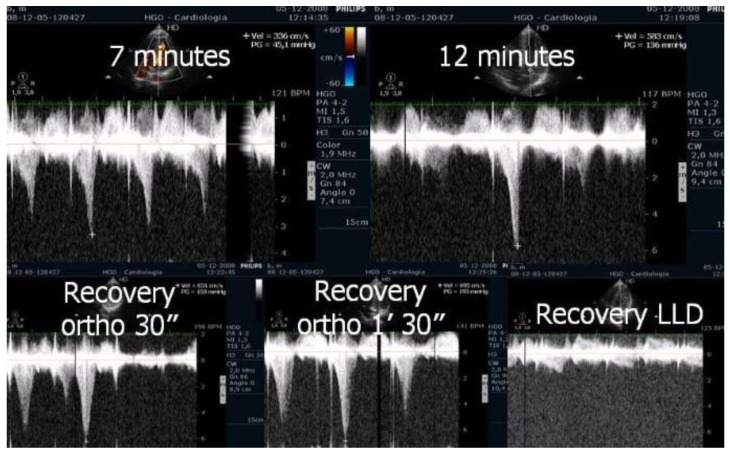
LVOT gradient in the various phases of exercise in the same symptomatic athlete [3].

**Figure 5:**
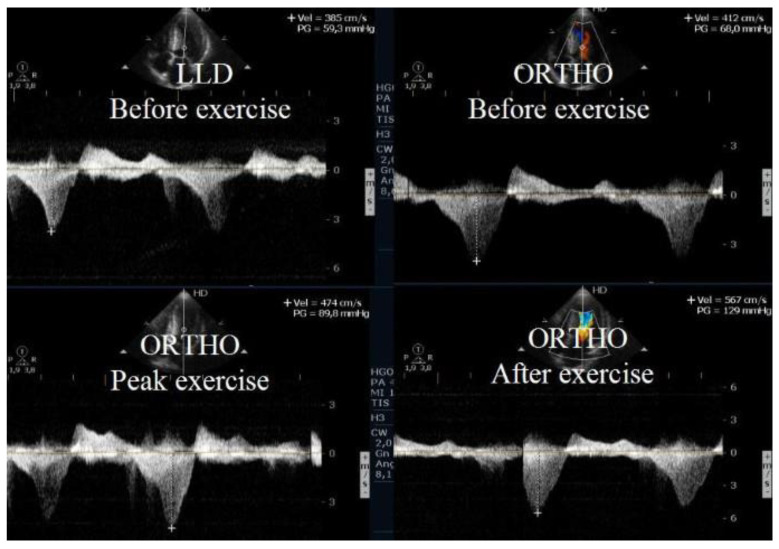
LVOT obstruction in one patient with HCM with a gradient that also increases in recovery in the orthostatic position [25].

The appearance of intraventricular gradients [17,22] is associated with morphological (Figure 2) determinants such as reduced LVOT index and reduced left ventricular diastolic volume, as observed in periods of increased heart rate. An anterior displacement of the posterior internal papillary muscle may also be involved in the development of intraventricular gradient and SAM of the mitral valve as described by other authors [23,24]. Immediately after exercise, the sudden LV preload reduction plus adrenergic response during this phase accounts for the LVOT gradient increase. However, the obstruction during the recovery period in the supine position as performed in some echocardiography labs is not fully representative of what occurs when the patient develops symptoms during day-to-day activities. In addition, in daily life, most patients who walk or run remain in a standing position after exercise and do not lie down. For this reason, post-exercise echocardiography after supine repositioning does not appear fully satisfactory. Accordingly, we [22,25] have highlighted that post-exercise (treadmill) evaluation of the LVOT gradient is more relevant with the patient in the standing position compared to the supine position. In our research, all patients with obstructive HCM developed increasing intra-ventricular gradients during orthostatic recovery than when compared to the supine position. To evaluate the clinical meaning of LVOT or MV obstruction, we consider exercise to be the only relevant stress since a high number of unselected patients develop obstruction with dobutamine, which is probably a pharmacologic effect [26]. Exercise echocardiography in the supine position is easier to perform; however, exercise in the orthostatic position and on the treadmill better reflects the patient’s normal activities and better replicates the clinical situations in which the symptoms usually occur. The orthostatic position, reducing the venous return (Figure 2), should be maintained after exercise, as a significant reduction in the LV volume has been shown to favor the appearance of the intraventricular obstruction under investigation (Figure 3, Figure 4 and Figure 5). Further, orthostatic evaluation is better than semi-supine bicycle exercise at evoking a dynamic intraventricular obstruction [27]. In this evaluation, two-dimensional mode echocardiographic images are selected from the parasternal window (in long-axis view and short-axis view) and apical window (four-chamber view and two-chamber view) in the orthostatic position at rest before exercise and during exercise, and at peak exercise; in the immediate after-exercise period; and during the recovery period in orthostatic position or in left lateral decubitus as appropriate. Image acquisition at peak exercise and immediately post-exercise can be carried out using a continuous image-capturing system. After the test, the loops with the best-quality image in each view are selected and archived. The acquired digitized images are then reviewed and compared in digital side-by-side quad-screen format with echocardiographic equipment. When relevant—e.g., for the detection and evaluation of intraventricular obstruction in hypertrophic cardiomyopathy (HCM), or in athletes with symptoms related to exercise—the patient remains in an orthostatic position after finishing the stress test, and echocardiography is obtained in this position. Although most groups perform exercise echocardiography during the recovery phase with the patient in lateral decubitus position, several groups have shown that peak exercise imaging is equally feasible and more accurate since regional wall motion abnormalities and dynamic gradients quickly disappear in the early recovery phase [28,29].

### 2.5. Outcome Data

The detection of LVOT or MV obstruction is crucial in the management of symptoms and the assessment of the risk of sudden death in patients with HCM. Two-dimensional and Doppler echocardiography during a Valsalva maneuver in the sitting and semi-supine positions—and then standing if no gradient is provoked—is recommended in all patients. Exercise stress echocardiography (SE) is recommended in symptomatic patients if bedside maneuvers fail to induce LVOT obstruction ≥ 30 mm Hg [4,30]. In HCM, a greater increase in dynamic gradients predicts a greater risk of sudden death and is used to help decide when to use a cardiac implantable device to protect against sudden death [4,30]. Taking into account Sherrid et al. [5], in which apical aneurysms in patients with apical HCM may hypothetically be caused by MV obstruction, exercise stress echocardiography may take on a more significant role in the future in the search for intraventricular gradients, leading to a more aggressive treatment strategy with beta blockers or more invasive therapies. We should remember that aneurysms are associated with a higher risk of ventricular arrhythmias and thrombosis, which consequently lead to a worse prognosis. Therefore, it is essential to prevent their development. In symptomatic athletes with LVOT or MV obstruction [15,22], the re-evaluation of the obstruction with exercise can also be used to monitor the effectiveness of treatment with a beta blocker. Additionally, it is important to clarify that one-third of previous exercise-related ischemia in patients with angiographically normal coronary arteries may be cases of LVOT or MV obstruction, which opens the door to effective treatment with beta blockers [31]. Based on our 20 years of experience with over 1300 patients systematically evaluated with upright treadmill testing, we note that the prevalence of a significant intraventricular (LVOT or MV) gradient in asymptomatic subjects is very rare, but becomes >30% in subjects, including athletes, who are symptomatic for dizziness, syncope, chest pain, or tiredness, without any other detectable etiology of symptoms, like valvular heart disease, diastolic dysfunction, or HCM [32]. Upright exercise imaging should be the standard approach in patients with an elusive cause of potentially life-threatening symptoms such as syncope, near syncope, or aborted sudden death [33,34] during exercise with an unknown cause, with or without HCM [1,33,34]. LVOT obstruction following mitral valve surgery and transcatheter mitral valve treatment can result from various mechanisms [35]. The acknowledgment of these, as well as their detection with appropriate imaging, namely exercise stress echocardiography, preferentially before and after the procedure, may be critical in achieving successful outcomes and also in understanding failures in the improvement of symptoms.

### 2.6. Tips and Tricks

Dynamic subaortic obstruction usually shows a typical late-peaking velocity curve, which frequently has a concave upward curve in the early systole (Figure 1). Some sources of error are known and should be minimized when measuring gradients, including malalignment of the jet and the ultrasound beam, and the recording of mitral regurgitation, which can be easily distinguished from LVOT or MV obstruction. In the majority of patients studied, the measurement of gradients is accurate, reproducible, highly feasible, and simple. The best way to detect LVOT or MV obstruction with exercise is to image the heart in the orthostatic position before exercise (usually easy), during exercise (difficult but feasible), and after exercise (usually easy), when the obstruction is greater. The standard approach of supine repositioning at the beginning of recovery after treadmill exercise misses a significant part of LVOT or MV obstruction with clinical relevance [25,27]. The value of orthostatic imaging is well known by clinical cardiologists who also routinely verify the appearance or increase in murmurs in HCM patients by performing cardiac auscultation in the orthostatic position or with the Valsalva maneuver. It is important to follow up on these patients and their conditions when they have symptoms. Upright exercise SE should be the standard approach in patients with relevant or severe symptoms related to exercise (syncope, near syncope, or aborted sudden death related to exercise) with or without HCM. It may also be useful to evaluate the presence of intraventricular obstruction induced by effort in patients with tiredness and normal ejection fraction. In the future, assessing pulmonary systolic arterial pressure should also be considered in characterizing severity [1,32,35,36,37,38] apart from the intraventricular obstruction. 

### 2.7. How to Report Gradients

A report on dynamic LVOT obstruction should provide a precise anatomic localization and quantification of the obstruction using color-guided, continuous-wave Doppler echocardiography in the apical five-chamber view. The gradient evaluation across the obstruction should be based on peak instantaneous gradient measurement, and the magnitude should be reported in millimeters of mercury [4,30,39]. The evolution of the LVOT obstruction during exercise SE in HCM should be observed carefully since it varies significantly at distinct levels of workload [25,40]. Assessment of LVOT obstruction should be performed and registered at least once at rest, at peak stress, and during the recovery phase. An echocardiographic report should describe, in detail, the anatomic and functional alterations in the mitral apparatus, which contribute to the development and severity of LVOT obstruction [41,42]. Hemodynamic and exercise parameters that can influence dynamic obstruction (e.g., prandial status, type, and protocol of stress, exercise time, maximum heart rate and workload achieved, or blood pressure response) are useful to communicate in parallel with LVOT or MV gradients [42]. Ongoing medical therapy should always be delineated, especially when drugs that affect the development and alteration of dynamic LVOT or MV obstruction are used [43]. It is particularly important to mention symptoms concurrently with SE findings, as they are determining factors in management decisions. However, if a patient shows no symptoms in the presence of provokable gradients, we should always question the true absence of symptoms or whether there is lifestyle adaptation. A reduction in LVOT obstruction during exertion, favoring a better clinical profile, should be highlighted and reported as a paradoxical hemodynamic response to exercise [44]. 

### 2.8. Clinical Guidelines and Recommendations

According to the more recent guidelines, stress-induced LVOT obstruction is a marker of a worse prognosis and has the potential to dictate major treatment decisions in HCM [4,30,39]. Exercise represents the most physiological and effective form of gradient provocation [27,42]. The peak gradient detected in the LVOT is indicative of obstruction if it reaches 30 mm Hg at rest and rises above 50 mm Hg with exercise. When associated with effort-related symptoms, the latter can be used as a threshold for invasive treatment [4,30]. The latest European (ESC) and American (AHA/ACC) guidelines for the diagnosis and treatment of patients with HCM agree that, in patients with symptomatic HCM who do not exhibit an outflow tract gradient of ≥50 mm Hg during standard echocardiographic evaluation, SE assessment should be performed to detect and quantify provocable LVOT obstruction [Class I, level of evidence (LOE) B]. Different suggestions are offered for asymptomatic individuals in the above-mentioned documents. The state-of-the-art AHA/ACC guidelines reveal that it can be beneficial to perform exercise SE in asymptomatic HCM patients without provocable obstruction (≥50 mm Hg) on standard transthoracic echocardiography (Class 2a, LOE C-LD), considering that it provides a comprehensive understanding of their individual pathophysiology. According to the ESC guidelines, the efficacy of exercise SE assessment in asymptomatic HCM patients with resting or bedside-provoked peak LVOT gradient < 50 mm Hg is less well established. Therefore, exercise SE may be considered only when the presence of an LVOT obstruction is relevant to lifestyle advice and decisions for medical treatment (Class IIb, LOE C). However, regardless of symptomatic status, LVOT obstruction assessment during exercise should be performed in every HCM patient without resting obstruction (≥50 mm Hg) who has a positive history of syncope [45]. According to the recent ESC guidelines on sports cardiology, all individuals with HCM who wish to participate in sports activity should have their LVOT gradient assessed after light exercise [34]. Moreover, outside HCM, the search for intraventricular gradients during exercise echocardiography can elucidate the hidden causes of unexplained dyspnea, exertional fatigue, post-exercise dizziness, or syncope in athletes or in patients with tiredness with preserved ejection fraction in the echocardiogram [32,38,42], which may affect future clinical management. 

## 3. Conclusions

In this study, it was determined that HCM patients develop increasing intraventricular gradients while in the orthostatic position, mainly during treadmill exercise and in the recovery period while sustaining the upright position. This evaluation can help us better understand the pathophysiology, symptomatology, and efficacy of different therapeutic modalities in this disease and should be routinely used in the assessment of HCM patients. It is essential for prognostication and is crucial in deciding on implantable cardioverter defibrillator implantation. Intraventricular obstruction has been reported in a significant number of patients with angina and normal coronary arteries, athletes with exercise-related symptoms, patients with unexplained tiredness and normal ejection fraction, and one athlete with aborted sudden death (Table 1).

In light of this published work, exercise stress echocardiography should be used to search intraventricular gradients in hypertrophic cardiomyopathy patients and in those patients beyond HCM with symptoms clearly related to exercise when all the other exams are normal. We hypothesize that beginning the diagnostic evaluation of the previous clinical scenarios with exercise stress echocardiography will lead to better outcomes and may be more cost-effective.

## Figures and Tables

**Table 1:** Studies with exercise stress echocardiography in HCM and other clinical situations in the references. DLVOTO—dynamic left ventricle outflow tract obstruction; EE—exercise echocardiography; ESC—European Society of Cardiology; HCM—hypertrophic cardiomyopathy; HOCM—hypertrophic obstructive cardiomyopathy; IVG—intraventricular gradient; LLD—left lateral decubitus; LVDVi—left ventricle diastolic volume index; LVOT—left ventricle outflow tract; LVOTi—left ventricle outflow tract index; RLVWT—relative left ventricle wall thickness; SAM—systolic anterior motion; SE—stress echocardiography.

Reference	Aims	Number of Patients	Type of Study	Results
in [1]	Assessment of beta-blocker therapy in athletes with exercise-induced intraventricular gradients (IVG).	35 symptomatic athletes	Clinical Trial	On therapy, during stress echocardiography (SE), there was a reduction in IVG at rest (35 off vs. 17 on beta blocker, *p* < 0.01), a decrease in IVG at peak exercise and immediately after (102 ± 34 mm Hg off vs. 69 ± 24 mm Hg on beta blocker, *p* < 0.01), peak heart rate (178 ± 15 bpm off vs. 157 ± 9 bpm on beta blocker), SAM of the mitral valve (24 off vs. 9 on beta blocker, *p* < 0.001), symptomatology during SE (17 off vs. 2 on beta blocker *p* < 0.001), and ST segment depression (13 off vs. 2 on the beta blocker, *p* < 0.001).
in [1]	Appraise the existence of IVG during exercise stress echocardiography in cardiac syndrome X.	91 patients with cardiac syndrome X	Research	IVG during upright exercise on a treadmill was developed in a statistically significant number of patients with cardiac X syndrome. This group of patients was characterized by being mainly of the male gender and younger than those who did not develop IVG. ST-segment downsloping during stress testing in patients without epicardial obstructive coronary disease appears to be correlated with the occurrence of IVG and SAM of the mitral valve on exertion. IVG and mitral valve SAM also seem to be associated with lower left ventricle outflow tract index (LVOTi), lower left ventricle diastolic volume index (LVDVi) and higher relative left ventricle wall thickness (RLVWT).
[18]	Define the incidence and predictors of dynamic left ventricle outflow tract obstruction (DLVOTO) in patients without hypertrophic obstructive cardiomyopathy (HOCM).	280 patients	Research	Echocardiographic signs of ischemia at peak exertion developed in 44%, and significant DLVOTO in 5% (13 patients). With multivariate analysis, it was found that independent predictors of significant DLVOTO at peak effort were increased septal wall thickness, higher systolic blood pressure, chordal SAM, younger age, and smaller left ventricle at end-systole. In at least 6 of the 13 patients, significant DLVOTO was a possible cause of symptoms and/or ischemia.
[19]	Describe LVOT velocities with maximal exercise in healthy youth.	50 children	Research	Peak LVOT velocities of ≥3 m/sec were developed by sixteen subjects (32%). Dynamic outflow tract Doppler pattern was evidenced in 12 of the 16 (75%) with elevated velocities, of whom eight had intracavitary narrowing on two-dimensional echocardiography. To conclude, the occurrence of significant exercise-induced LVOT velocities may be considered a normal physiologic finding in healthy youth.
in [1]	To study the prevalence of exercise IVGs in athletes whose preparticipation cardiovascular screening result for sports practice was positive according to ESC guidelines, and evaluate exercise echocardiography on the detection of IVGs.	139 athletes	Comparative study	SAM of the mitral valve was observed in 33 of the 52 athletes in the group with IVG, and in none of the athletes in the group without IVG. The results highlight that those athletes with exercise-induced symptoms and/or ischemia-like electrocardiographic signs are often associated with significant IVG, developing in the absence of regional wall motion abnormalities. IVG was also more evident at the beginning of exercise during treadmill upright imaging.
in [1]	Evaluate IVGs with echocardiography during treadmill exercise and post-exercise in the upright position in patients with HCM.	17 HCM	Research	Three patients with non-obstructive HCM at rest developed IVGs during exercise. One patient developed this gradient only during orthostatic recovery. The mean IVG in left lateral decubitus (LLD) was 49 ± 4 mm Hg; in orthostatic position it was 62 ± 29 mm Hg (*p* < 0.001 versus in LLD); at peak exercise it was 83 ± 35 mm Hg (*p* < 0.001 versus supine rest); during recovery it was 96 ± 35 mm Hg (*p* < 0.001 versus peak exercise)
[27]	As various types of exercise have different consequences on peripheral vascular circulation, this study sought to compare upright treadmill exercise echocardiography (EE) to semi-supine bicycle EE in maximum provoked LVOTO in HCM patients.	23 HCM	Comparative study	This study demonstrates that, when compared to the semi-supine bicycle EE, the treadmill is better for ascertaining the maximum LVOT gradient in HCM.
[40]	The relation between functional capacity and exercise-induced LVOT obstruction in HCM is incompletely defined. The time course of the provoked gradients and the relation to exercise performance were assessed.	74 HCM	Research	In patients with non-obstructive HCM at rest, the earlier onset of LVOT gradients during treadmill exercise was associated with impaired exercise performance. These findings have yielded insights into the determinants of functional impairment in HCM and support the potential value of exercise echocardiography in the clinical evaluation of patients with HCM.

## Data Availability

Data can be made available on written request.

## Abbreviations

ACC/AHA	American College of Cardiology/American Heart Association
ESC	European Society of Cardiology
HCM	Hypertrophic cardiomyopathy
LOE	Level of evidence
LVOT	Left ventricular outflow tract
MV	Mid-ventricular
SAM	Systolic anterior motion
SE	Stress echocardiography

## References

[1] Cotrim C.A., Café H., João I., Cotrim N., Guardado J., Cordeiro P., Cotrim H., Baquero L. (2022). Exercise stress echocardiography: Where are we now?. World J. Cardiol..

[2] Peteiro J., Bouzas-Mosquera A. (2010). Exercise echocardiography. World J. Cardiol..

[3] Petkow Dimitrow P., Cotrim C., Cheng T.O. (2014). Need for a standardized protocol for stress echocardiography in provoking subaortic and valvular gradient in various cardiac conditions. Cardiovasc. Ultrasound..

[4] Elliott P.M., Anastasakis A., Borger M.A., Borggrefe M., Cecchi F., Charron P., Hagege A.A., Lafont A., Limongelli G., Mahrholdt H. (2014). 2014 ESC Guidelines on diagnosis and management of hypertrophic cardiomyopathy: The Task Force for the Diagnosis and Management of Hypertrophic Cardiomyopathy of the European Society of Cardiology (ESC). Eur. Heart J..

[5] Sherrid M.V., Bernard S., Tripathi N., Patel Y., Modi V., Axel L., Talebi S., Ghoshhajra B.B., Sanborn D.Y., Saric M. (2023). Apical Aneurysms and Mid-Left Ventricular Obstruction in Hypertrophic Cardiomyopathy. JACC Cardiovasc. Imaging.

[6] Guerra M., Sampaio F., Brás-Silva C., Leite-Moreira A.F. (2011). Left intraventricular diastolic and systolic pressure gradients. Exp. Biol. Med..

[7] Firstenberg M.S. (2013). Intraventricular pressure gradients: The often-ignored question of how and why does the ventricle suck. Exp. Physiol..

[8] Courtois M., Kovács S.J., Ludbrook P.A. (1988). Transmitral pressure-flow velocity relation. Importance of regional pressure gradients in the left ventricle during diastole. Circulation.

[9] Yotti R., Bermejo J., Bento Y., Antoranz J.C., Desco M.M., Rodríguez-Pérez D., Cortina C., Mombiela T., Barrio A., Elízaga J. (2011). Noninvasive estimation of the rate of relaxation by analysis of intraventricular pressure gradients. Circ. Cardiovasc. Imaging.

[10] Smiseth O.A., Steine K., Sandbaek G., Stugaard M., Gjolberg T. (1998). Mechanics of intraventricular filling: Study of LV early diastolic pressure gradients and flow velocities. Am. J. Physiol..

[11] Pasipoularides A., Murgo J.P., Miller J.W., Craig W.E. (1987). Nonobstructive left ventricular ejection pressure gradients in man. Circ. Res..

[12] Falsetti H.L., Verani M.S., Chen C.J., Cramer J.A. (1980). Regional pressure differences in the left ventricle. Cathet. Cardiovasc. Diagn..

[13] Butler C.K., Wong A.Y., Armour J.A. (1988). Systolic pressure gradients between the wall of the left ventricle, the left ventricular chamber, and the aorta during positive inotropic states: Implications for left ventricular efficiency. Can. J. Physiol. Pharmacol..

[14] Yotti R., Bermejo J., Desco M.M., Antoranz J.C., Rojo-Alvarez J.L., Cortina C., Allué C., Rodríguez-Abella H., Moreno M., García-Fernández M.A. (2005). Doppler-derived ejection intraventricular pressure gradients provide a reliable assessment of left ventricular systolic chamber function. Circulation.

[15] Cabrera-Bueno F., García-Pinilla J.M., Gómez-Doblas J.J., Montiel-Trujillo A., Rodríguez-Bailón I., de Teresa-Galván E. (2007). Beta-blocker therapy for dynamic left ventricular outflow tract obstruction induced by exercise. Int. J. Cardiol..

[16] Yotti R. (2004). Qué significado tiene un gradiente de presión intraventricular sistólico durante el ejercicio? [What is the relevance of an intraventricular ejection pressure gradient induced by exercise?]. Rev. Esp. Cardiol..

[17] Cabrera Bueno F., Rodríguez Bailón I., López Salguero R., Gómez Doblas J.J., Pérez Cabeza A., Peña Hernández J., Domínguez Franco A., Morcillo Hidalgo L., de Teresa Galván E. (2004). Obstrucción dinámica intraventricular izquierda inducida por esfuerzo [Dynamic left ventricular outflow tract obstruction induced by exercise]. Rev. Esp. Cardiol..

[18] Zywica K., Jenni R., Pellikka P.A., Faeh-Gunz A., Seifert B., Attenhofer Jost C.H. (2008). Dynamic left ventricular outflow tract obstruction evoked by exercise echocardiography: Prevalence and predictive factors in a prospective study. Eur. J. Echocardiogr..

[19] Wittlieb-Weber C.A., Cohen M.S., McBride M.G., Paridon S.M., Morrow R., Wasserman M., Wang Y., Stephens P. (2013). Elevated left ventricular outflow tract velocities on exercise stress echocardiography may be a normal physiologic response in healthy youth. J. Am. Soc. Echocardiogr..

[20] Tozzi R.J. (2014). Left ventricular outflow tract velocities-all in context. J. Am. Soc. Echocardiogr..

[21] Grose R., Maskin C., Spindola-Franco H., Yipintsoi T. (1981). Production of left ventricular cavitary obliteration in normal man. Circulation.

[22] Saeed S., Vegsundvåg J. (2021). Usefulness of Stress Echocardiography in Assessment of Dynamic Left Ventricular Obstructions: Case Series and Review of the Literature. Cardiology.

[23] Levine R.A., Vlahakes G.J., Lefebvre X., Guerrero J.L., Cape E.G., Yoganathan A.P., Weyman A.E. (1995). Papillary muscle displacement causes systolic anterior motion of the mitral valve. Experimental validation and insights into the mechanism of subaortic obstruction. Circulation.

[24] Queiróz e Melo J., Canada M., Neves J., Ferreira M.M., de Sousa J.S., Ribeiras R., Rebocho M.J., de Santos J.C., Seabra-Gomes R. (1996). Insercão anómala de músculos papilares mitrais na miocardiopatia hipertrófica obstrutiva. A propósito de dois casos [Anomalous insertion of mitral papillary muscles in obstructive hypertrophic myocardiopathy. Report of 2 cases]. Rev. Port. Cardiol..

[25] Miranda R., Cotrim C., Cardim N., Almeida S., Lopes L., Loureiro M.J., Simões O., Cordeiro P., Fazendas P., João I. (2008). Evaluation of left ventricular outflow tract gradient during treadmill exercise and in recovery period in orthostatic position, in patients with hypertrophic cardiomyopathy. Cardiovasc. Ultrasound..

[26] Pellikka P.A., Oh J.K., Bailey K.R., Nichols B.A., Monahan K.H., Tajik A.J. (1992). Dynamic intraventricular obstruction during dobutamine stress echocardiography. A new observation. Circulation.

[27] Reant P., Dufour M., Peyrou J., Reynaud A., Rooryck C., Dijos M., Vincent C., Cornolle C., Roudaut R., Lafitte S. (2018). Upright treadmill vs. semi-supine bicycle exercise echocardiography to provoke obstruction in symptomatic hypertrophic cardiomyopathy: A pilot study. Eur. Heart J. Cardiovasc. Imaging.

[28] Peteiro J., Garrido I., Monserrat L., Aldama G., Calviño R., Castro-Beiras A. (2004). Comparison of peak and postexercise treadmill echocardiography with the use of continuous harmonic imaging acquisition. J. Am. Soc. Echocardiogr..

[29] Peteiro J., Bouzas-Mosquera A., Broullón F.J., Garcia-Campos A., Pazos P., Castro-Beiras A. (2010). Prognostic value of peak and post-exercise treadmill exercise echocardiography in patients with known or suspected coronary artery disease. Eur. Heart J..

[30] Ommen S.R., Mital S., Burke M.A., Day S.M., Deswal A., Elliott P., Evanovich L.L., Hung J., Joglar J.A., Kantor P. (2020). 2020 AHA/ACC Guideline for the Diagnosis and Treatment of Patients With Hypertrophic Cardiomyopathy: Executive Summary: A Report of the American College of Cardiology/American Heart Association Joint Committee on Clinical Practice Guidelines. Circulation.

[31] Sherrid M.V., Pearle G., Gunsburg D.Z. (1998). Mechanism of benefit of negative inotropes in obstructive hypertrophic cardiomyopathy. Circulation.

[32] Cotrim C., Café H., Gonçalves I., Guardado J., Cotrim N., Cordeiro P., Feliciano J., Baquero L., Picano E. (2021). Upright exercise stress echocardiography may unmask dynamic left ventricular obstruction also beyond hypertrophic cardiomyopathy. Eur. Heart J..

[33] Cotrim C.A., Cotrim N., Guardado J.H., Baquero L. (2023). Exercise-Induced intraventricular gradients as a potential cause of Sudden Cardiac Death. Cureus.

[34] Pelliccia A., Sharma S., Gati S., Bäck M., Börjesson M., Caselli S., Collet J.P., Corrado D., Drezner Prescott E., Roos-Hesselink J.W. (2021). 2020 ESC Guidelines on sports cardiology and exercise in patients with cardiovascular disease. Eur. Heart J..

[35] YYSilbiger J.J., Lee S., Christia P., Perk G. (2019). Mechanisms, pathophysiology, and diagnostic imaging of left ventricular outflow tract obstruction following mitral valve surgery and transcatheter mitral valve replacement. Echocardiography.

[36] Ha J.W., Andersen O.S., Smiseth O.A. (2020). Diastolic Stress Test: Invasive and Noninvasive Testing. JACC Cardiovasc. Imaging.

[37] Shim C.Y., Kim S.A., Choi D., Yang W.I., Kim J.M., Moon S.H., Lee H.J., Park S., Choi E.Y., Chung N. (2011). Clinical outcomes of exercise-induced pulmonary hypertension in subjects with preserved left ventricular ejection fraction: Implication of an increase in left ventricular filling pressure during exercise. Heart.

[38] Alhaj E.K., Kim B., Cantales D., Uretsky S., Chaudhry F.A., Sherrid M.V. (2013). Symptomatic exercise-induced left ventricular outflow tract obstruction without left ventricular hypertrophy. J. Am. Soc. Echocardiogr..

[39] Turvey L., Augustine D.X., Robinson S., Oxborough D., Stout M., Smith N., Harkness A., Williams L., Steeds R.P., Bradlow W. (2021). Transthoracic echocardiography of hypertrophic cardiomyopathy in adults: A practical guideline from the British Society of Echocardiography. Echo Res. Pract..

[40] Nistri S., Olivotto I., Maron M.S., Grifoni C., Baldini K., Baldi M., Sgalambro A., Cecchi F., Maron B.J. (2010). Timing and significance of exercise-induced left ventricular outflow tract pressure gradients in hypertrophic cardiomyopathy. Am. J. Cardiol..

[41] Nagueh S.F., Bierig S.M., Budoff M.J., Desai M., Dilsizian V., Eidem B., Goldstein S.A., Hung J., Maron M.S., Ommen S.R. (2011). American Society of Echocardiography clinical recommendations for multimodality cardiovascular imaging of patients with hypertrophic cardiomyopathy: Endorsed by the American Society of Nuclear Cardiology, Society for Cardiovascular Magnetic Resonance, and Society of Cardiovascular Computed Tomography. J. Am. Soc. Echocardiogr..

[42] Lancellotti P., Pellikka P.A., Budts W., Chaudhry F.A., Donal E., Dulgheru R., Edvardsen T., Garbi M., Ha J.W., Kane G.C. (2017). The clinical use of stress echocardiography in non-ischaemic heart disease: Recommendations from the European Association of Cardiovascular Imaging and the American Society of Echocardiography. J. Am. Soc. Echocardiogr..

[43] Ammirati E., Contri R., Coppini R., Cecchi F., Frigerio M., Olivotto I. (2016). Pharmacological treatment of hypertrophic cardiomyopathy: Current practice and novel perspectives. Eur. J. Heart Fail..

[44] Lafitte S., Reant P., Touche C., Pillois X., Dijos M., Arsac F., Peyrou J., Montaudon M., Ritter P., Roudaut R. (2013). Paradoxical response to exercise in asymptomatic hypertrophic cardiomyopathy: A new description of outflow tract obstruction dynamics. J. Am. Coll. Cardiol..

[45] Brignole M., Moya A., de Lange F.J., Deharo J.C., Elliott P.M., Fanciulli A., Fedorowski A., Furlan R., Kenny R.A., Martín A. (2018). 2018 ESC Guidelines for the diagnosis and management of syncope. Eur. Heart J..

